# Predicting prognosis of patients with hepatitis B virus-related acute-on-chronic liver failure from longitudinal ultrasound images using a multi-task deep learning approach

**DOI:** 10.1080/07853890.2025.2551819

**Published:** 2025-08-26

**Authors:** Xingzhi Huang, Songsong Yuan, Aiyun Zhou, Xinchun Yuan, Yaohui Li, Yufan Kuang, Pan Xu

**Affiliations:** ^a^ Department of Ultrasound, The First Affiliated Hospital, Jiangxi Medical College, Nanchang University, Nanchang, Jiangxi, China; ^b^ Department of Infectious Disease, The First Affiliated Hospital, Jiangxi Medical College, Nanchang University, Nanchang, Jiangxi, China; ^c^ Key Laboratory of Infectious Diseases, The First Affiliated Hospital, Jiangxi Medical College, Nanchang University, Nanchang, Jiangxi, China

**Keywords:** Acute-on-chronic liver failure, hepatitis B virus, Siamese network, multi-task network, prognosis

## Abstract

**Background:**

Individualized risk stratification in hepatitis B virus-related acute-on-chronic liver failure (HBV-ACLF) remains challenging. This study aimed to develop and validate a multi-task deep learning model using longitudinal liver ultrasound images for prognosis prediction.

**Methods:**

A total of 372 HBV-ACLF patients were retrospectively enrolled, and baseline (T0) and 5 days post-admission (T1) liver ultrasound images, clinical data, and 30-day outcome (survival/mortality) were collected. A Siamese U-net deep learning model (Siamese U-Net) was trained to automatically segment the liver region and predict 30-day mortality using longitudinal liver ultrasound images from the training cohort (*n* = 290). The model output and clinical predictors were integrated into a combined model *via* Cox regression, with a clinical model developed for comparison. Model performance was evaluated for segmentation and prediction in the validation cohort (*n* = 82).

**Results:**

Siamese U-Net-generated masks achieve Dice Coefficients of 0.912 and 0.913 against manual delineation for T0 and T1 images in the validation cohort. The Siamese U-Net significantly outperformed the clinical model (*p* < 0.01), achieving a C-index of 0.795 and an AUC of 0.851 in the validation cohort. Calibration curves and decision curve analyses showed superior calibration and clinical utility. The combined model achieved a C-index of 0.834 and an AUC of 0.892, marginally improving the Siamese U-Net (*p* > 0.05) but significantly enhancing the clinical model (*p* < 0.01) in the validation cohort.

**Conclusions:**

The Siamese U-Net emerges as a promising tool in predicting prognosis for HBV-ACLF, thereby enhancing clinical decision-making and improving patient outcomes.

## Introduction

Acute-on-chronic liver failure (ACLF) is a clinical syndrome characterized by acute deterioration of pre-existing chronic liver disease, with a high short-term risk of multi-organ failure and mortality [1,2]. In the Asia-Pacific and African regions, hepatitis B virus (HBV) is the predominant cause, and HBV‑ACLF patients experience higher rates of infection, liver and coagulation failure, and mortality [3,4]. While some patients recover with standard therapy, others require intensive care unit support and liver transplantation [5,6]. Therefore, early and reliable prognostic models are essential for improving outcomes and optimizing the allocation of scarce resources such as liver transplants.

Risk stratification in chronic liver disease has traditionally relied on clinical data; however, these models demonstrate only moderate discrimination in HBV-ACLF in prior studies [7–9]. Increasing research has leveraged machine learning to develop predictive models grounded exclusively in clinical parameters, yet such models are computationally complex and rely on diverse clinical data [7,10]. Medical imaging of the liver has emerged as a valuable adjunct for assessing disease severity, since changes in hepatic morphology, parenchymal texture and perfusion dynamics reflect the progression of liver failure [11,12]. Imaging biomarkers facilitate prognostic prediction for HBV-ACLF across dimensions distinct from clinical indicators, and when integrated with clinical data, they harness such multi-modal synergy to enhance predictive accuracy [13–15]. Ultrasound proves well-suited for liver assessment, with its radiomics and deep learning having achieved fibrosis-staging accuracy on par with biopsy, and radiomic models further effectively stratify risk of post-hepatectomy liver failure in hepatocellular carcinoma patients and short-term mortality in HBV-ACLF patients [16–18].

The course of HBV-ACLF is rapid and volatile, with mortality risk dynamically changing as the disease progresses rapidly. Thus, dynamic analysis of clinical data has been proven superior to baseline parameters [19,20]. We hypothesize that predictive models incorporating longitudinal liver ultrasound images will outperform single-timepoint image-based models. With the increasing demand for mining temporal information, deep learning, particularly the Siamese network, has emerged as a prominent approach [21]. Meanwhile, multi-task deep learning networks can achieve positive feedback between related tasks, thereby enhancing the overall performance of the model [22–24]. Notably, Siamese architecture offers the ability to model temporal changes *via* element-wise operations for handling longitudinal data, while facilitating joint optimization of interdependent tasks, thus rendering it well-suited for applications demanding anatomical precision and prognostic accuracy across limited time points. Previous studies have demonstrated that multi-task deep learning models trained on tumor images acquired before and during neoadjuvant chemoradiotherapy exhibit superior predictive performance for treatment outcomes compared with single-timepoint or single-task models [23,24].

Here, we developed and validated a Siamese multi-task deep learning framework based on baseline and early-treatment liver ultrasound images of HBV-ACLF patients, which can simultaneously segment liver region and predict short-term prognosis. Through the integration of multi-scale features and dynamic information, the model emerges as a novel tool for delivering more precise prognostic predictions.

## Methods

This retrospective study conformed to the Declaration of Helsinki and received approval from the Institutional Review Board (IRB) of the First Affiliated Hospital of Nanchang University (2025CDYFYYLK01-022). The IRB granted a waiver of informed consent given the retrospective nature of the study, while all patient data (including ultrasound images and clinical records) were de-identified prior to analysis, thereby safeguarding patient confidentiality.

### Patients

Data of patients with HBV-ACLF between January 2021 and December 2022 were collected from the Department of Infectious Diseases at our institution. The inclusion criteria were: 1) meeting the Asian Pacific association for the study of the liver (APASL) ACLF diagnostic criteria (See Supplementary S1.1 for the definition) [2], which is the standard utilized for ACLF diagnosis at our institution; 2) positive HBsAg for more than 6 months; 3) cirrhosis; 4) undergoing liver ultrasound on the day of admission (baseline/T0) and 5 days later (early treatment phase/T1). Exclusion criteria included: 1) alcohol-related, autoimmune, toxic, or drug-induced liver injury; 2) concurrent hepatocellular carcinoma or extrahepatic malignant diseases; 3) pregnancy; 4) lost to follow-up or underwent liver transplantation during the follow-up period; 5) unqualified ultrasound image quality, such as artifacts or intestinal gas interference; 6) age under 18 years.

In total, 372 patients were included and grouped by inclusion time, with 290 patients from January 2021 to June 2022 comprising the training cohort and 82 patients from July 2022 to December 2022 comprising the validation cohort. The 30-day mortality rates were 30.0% (87/290) and 28.0% (23/82), respectively. The detailed flowchart for patient enrollment is shown in Fig. S1. During hospitalization, patients were provided with standard antiviral therapy, supportive care, and monitoring, with treatment adjusted based on each patient’s condition and response [2].

### Clinical data

Baseline characteristics collected comprised age, sex, and mean arterial pressure. Laboratory tests were enrolled including hepatitis B virus (HBeAg status and HBV DNA levels), blood counts, electrolytes, liver and kidney function, coagulation, and Alpha-Fetoprotein. The organ involvement definition is provided in Supplementary S1.2. The 30-day outcome of each patient was recorded, which was defined as all-cause mortality attributable to HBV-ACLF or its complications. Follow-up procedures were initiated on the date of admission, which comprised electronic medical record system review and telephone interviews.

### Longitudinal ultrasound image acquisition and processing

Liver ultrasound examinations were performed by experienced radiologists using Supersonic Aixplorer, Philips EPIQ 7, and WISONIC Clover 60/Clover 90 ultrasound machines. The right intercostal oblique section (the section of the first porta hepatis) was selected as the target section. For each patient, longitudinal images were obtained by the same radiologist using the same machine. The text and icons about equipment and parameters were cropped from the original images, resulting in rectangular images and resized to 256x256x1 pixels before being used to train the deep learning network. The liver region of interest (ROI) was manually outlined by another three experienced radiologists (X.Z.H., P.X., and A.Y.Z., with 8–25 years of experience) blinded to the patients’ outcome, using ITK-SNAP software (version 3.8). Supplementary S2 provides further details on the segmentation process.

### Development of the deep learning model

**Figure 1.**
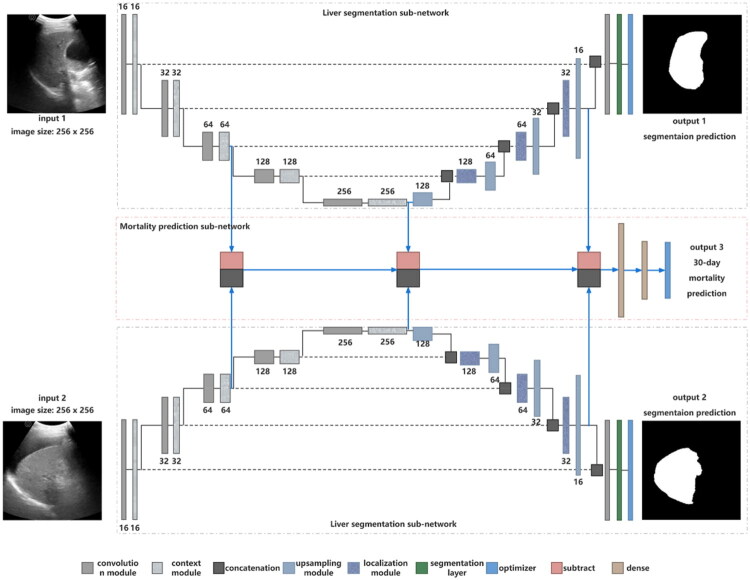
Detailed illustration of the Siamese U-Net architecture. The Siamese U-Net, composed of dual modified U-nets, processes longitudinal ultrasound images and masks at admission (T0) and 5 days post-admission (T1). Each U-net, via encoder-decoder paths with skip connections, generates automated liver segmentation masks for T0 and T1. The model simultaneously integrates multi-scale features from intermediate/bottleneck layers of the U-nets and dynamic changes between T0 and T1 to output 30-day mortality probability.

In this study, a Siamese U-net deep learning model (Siamese U-Net) was constructed for simultaneously performing segmentation and prediction. The model takes the liver ultrasound images and masks at T0 and T1 as inputs, and outputs the automatically delineated liver masks and the probability values of the 30-day mortality. The network architecture is shown in Figure 1 includes two sub-networks: the segmentation sub-network extracts features and segments the liver region using two modified U-nets with encoder-decoder paths and skip connections, and the prediction sub-network analyzes prognostic information from multi-scale features and liver dynamic changes by concatenating imaging features from the intermediate layer in the contracting path, bottleneck layer of the U-net, and elementwise summation module at the end.

A multi-task loss function was used to train the model, combining two loss components: focal loss addressed class imbalance between survival and mortality, and a combination of Binary Cross-Entropy loss and Dice loss mitigated the foreground-background imbalance in liver ultrasound images [25,26]. Strategies to mitigate overfitting and acquisition variability during training included data augmentation, dropout, batch normalization, and early stopping. Training was conducted using data from the training cohort on a NVIDIA GeForce RTX 4090, leveraging the Keras framework with a TensorFlow backend. Network architecture, loss functions, and training process details are provided in Supplementary S3.

### Deep learning model Ablation analysis

To validate the necessity of individual components in the proposed network, a series of ablation experiments were conducted by simplifying or modifying the network. First, we removed half of the Siamese U-Net architecture and trained multi-task networks (T0/T1-MT-Net) using single-timepoint images (Fig. S2). Second, we replaced the U-net architecture in the Siamese U-Net with ResNet-101, constructing a single-task model (Siamese ResNet-101) trained on longitudinal images (Fig. S3). In addition, we removed both the decoders and the dynamic branches, resulting in a single-scale feature-based model termed the Siamese encoder-Net (Fig. S4).

### Integration with clinical parameters

We utilized univariate and multivariate Cox regression analyses to identify independent clinical predictors for 30-day mortality in the training cohort, and the hazard ratio (HR) with 95% confidence interval (CI) was calculated. Initially, univariate Cox regression was performed on all candidate variables, with variables achieving *p* < 0.05 advanced to a stepwise multivariate model to account for potential confounding factors. Variables achieving statistical significance (*p* < 0.05) in the multivariate model were incorporated into two prognostic models *via* multivariate Cox regression: (1) a clinical model based solely on these variables, and (2) a combined model integrating these clinical parameters with Siamese U-Net-derived predictions.

### Model performance evaluation metrics

First, to evaluate its segmentation performance, we calculated the Dice coefficient between the model’s output and manual delineations.

For prediction performance, we determined the optimal cutoff point by maximizing the Youden’s index on the receiver operating characteristic (ROC) curve. Predictive discrimination was evaluated using sensitivity, specificity, accuracy, concordance index (C-index), the area under the curve (AUC), and Kaplan-Meier curves. Calibration was assessed with the calibration curve and integrated Brier score [27]. Decision curve analysis (DCA) was performed to quantify the clinical usefulness [28]. Predictive accuracy was compared between the Siamese U-Net and its ablated variants in the ablation analysis. Integrated discrimination improvement (IDI), net reclassification improvement (NRI), and relative prediction error reduction were used to quantify the additive value of Siamese U-Net predictions over clinical data alone.

**Figure 2.**
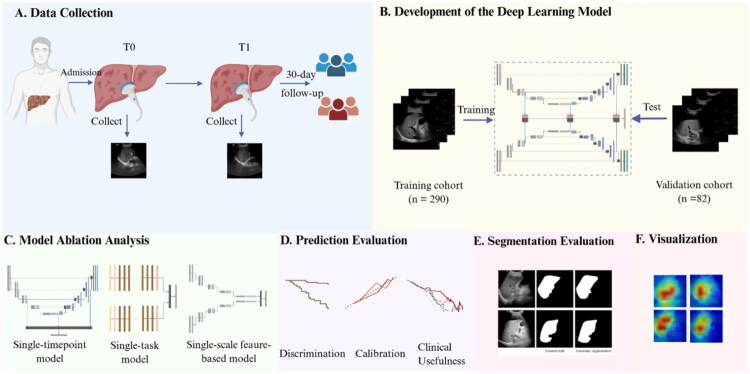
The overall design of the study. (A) Collection of liver ultrasound images at admission (T0) and 5 days post-admission (T1), along with 30-day prognoses of HBV-ACLF patients. (B) Development and validation of a deep learning model for prognosis prediction and liver region segmentation: training cohort data were used to construct the model, then tested in the validation cohort. (C) Ablation experiments were performed to validate the importance of individual components in the proposed network. (D-F) Model performance was assessed with respect to outcome prediction, segmentation, and visualization.

To understand the decision-making process of the Siamese U-Net in predicting prognosis, we utilized gradient-weighted class activation mapping (Grad-CAM) to visualize the regions on ultrasound images that contributed most to the model’s predictions [29]. The overall workflow of this study is illustrated in Figure 2.

### Statistical analysis

Statistical analysis was performed using R (version 4.2.2). Quantitative data were expressed as median and interquartile range (IQR) and compared by Mann-Whitney U tests. Categorical data were presented as frequency (percentage) and compared by Chi-square or Fisher’s exact tests. Kaplan-Meier curves were compared using a log-rank test. Differences in the AUCs and C-indices were evaluated using the DeLong test and Monte Carlo simulation method, respectively [30]. All statistical tests were two-sided, and *p* < 0.05 was considered statistically significant.

## Results

### Patient characteristics

Patient characteristics for the training and validation cohorts are presented in Table 1. No statistically significant differences were observed between both cohorts (*p* > 0.05). Non-survivors had significantly higher rates of liver, renal, and coagulation failure, along with elevated levels of total bilirubin, creatinine, and international normalized ratio (INR) compared with survivors (*p* < 0.05).

**Table 1.** Patient characteristics in the training and validation cohorts.

Patient characteristics	Training cohort			Validation cohort			*p*#
	Survivors	Non-Survivors	p*	Survivors	Non-survivors	p*	
	(*n* = 203)	(*n* = 87)		(*n* = 59)	(*n* = 23)		
Age (years)	48.0 (43.0–57.0)	51.0 (41.5–62.5)	0.117	49.0 (44.0–59.0)	50.0 (39.5–56.0)	0.386	0.996
Female (%)	48 (23.6)	18 (20.7)	0.691	12 (20.3)	5 (21.7)	1.000	0.811
MAP (mmHg)	86.0 (79.8–95.8)	86.0 (76.5–95.5)	0.464	84.7 (78.3–94.2)	85.0 (72.2–96.3)	0.602	0.309
**Organ failure**							
Liver (%)	128 (63.1)	80 (92.0)	<0.001	44 (74.6)	22 (95.7)	0.017	0.147
Kidney (%)	4 (2.0)	11 (12.6)	<0.001	2 (3.4)	4 (17.4)	0.049	0.426
Coagulation (%)	20 (9.9)	26 (29.9)	<0.001	7 (11.9)	8 (34.8)	0.025	0.722
CNS (%)	3 (1.5)	11 (12.6)	<0.001	1 (1.7)	1 (4.3)	0.485	1.000
Respiratory system (%)	0 (0)	0 (0)	–	0 (0)	1 (4.3)	0.281	0.220
Circulatory system (%)	4 (2.0)	4 (4.6)	0.247	1 (1.7)	1 (4.3)	0.485	1.000
**Laboratory data**							
HBeAg positive (%)	65 (32.0)	26 (29.9)	0.825	20 (33.9)	12 (52.2)	0.203	0.243
HBV DNA (log10 IU/mL)	4.8 (2.7–6.3)	4.3 (0.0–5.9)	0.144	4.1 (0.8–5.8)	5.6 (1.9–7.7)	0.072	0.855
WBC (10^9/L)	5.9 (4.4–7.5)	6.5 (5.1–9.8)	0.012	6.4 (4.3–7.9)	7.3 (5.5–10.0)	0.050	0.442
Albumin (g/dL)	3.0 (2.7–3.3)	3.0 (2.7–3.3)	0.487	3.2 (2.8–3.4)	3.2 (2.9–3.3)	0.745	0.063
Total bilirubin (mg/dL)	15.5 (9.9–22.0)	21.7 (15.9–26.4)	<0.001	17.5 (12.7–23.4)	23.5 (17.1–28.3)	0.005	0.076
Creatinine (mg/dL)	0.7 (0.6–0.9)	0.8 (0.6–1.1)	0.026	0.7 (0.6–0.8)	0.9 (0.6–1.3)	0.015	0.924
Serum sodium (mmol/L)	136.8 (133.9–139.6)	137.0 (134.3–139.0)	0.906	136.2 (134.2–139.0)	136.0 (130.7–138.4)	0.267	0.106
ALT (IU/L)	231.6 (66.3–630.2)	232.3 (85.2–690.0)	0.664	339.0 (119.4–687.0)	623.9 (104.2–915.7)	0.591	0.254
AST (IU/L)	204.0 (99.2–479.6)	217.9 (99.5–510.3)	0.607	237.8 (124.5–349.7)	336.1 (110.2–563.7)	0.595	0.636
INR	1.8 (1.6–2.1)	2.0 (1.7–2.6)	<0.001	1.8 (1.5–2.0)	2.0 (1.9–2.9)	<0.001	0.720
AFP (ng/mL)	33.8 (7.4–92.2)	58.1 (11.6–147.1)	0.078	49.0 (16.6–129.6)	46.2 (8.5–257.4)	0.824	0.276

Data are expressed as the median (interquartile range) or n (%).

*: *p* value for the comparison between survivors and non-survivors within the cohort. #: *p* value for the comparison between the training and validation cohorts.

Abbreviations: AFP, alpha-fetoprotein; ALT, alanine aminotransferase; AST, aspartate aminotransferase; HBV, hepatitis B virus; INR, international normalized ratio; MAP, mean arterial pressure; MELD, model for end-stage liver disease; PLT, Platelet count; WBC, white blood cell.

### Segmentation performance of the deep learning model

**Figure 3.**
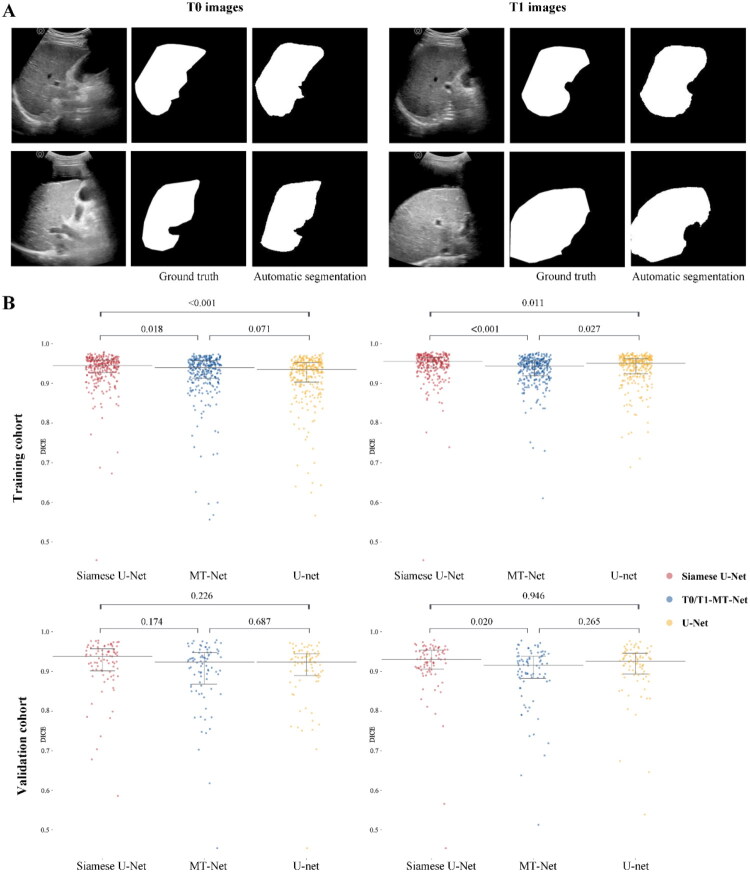
Segmentation performance of the Siamese U-Net. (A) T0 and T1 images of two patients. From left to right: origin ultrasound, ground truth and segmentation result. (B) Comparison of Dice coefficient (DICE) in the training and validation cohorts by three segmentation methods Siamese U-Net, T0/T1-MT-Net and U-Net.

Dice coefficients demonstrated that the liver masks predicted by the Siamese U-Net exhibited high similarity with manually delineated ROIs. In the validation cohort, Dice coefficients were 0.912 (95% CI 0.897–0.927) for T0 images and 0.913 (95% CI 0.897–0.930) for T1 images. Furthermore, the Siamese U-Net, T0/T1-MT-Net, and U-net models exhibited comparable segmentation performance (Figure 3).

### Predictive performance of the deep learning model

**Figure 4.**
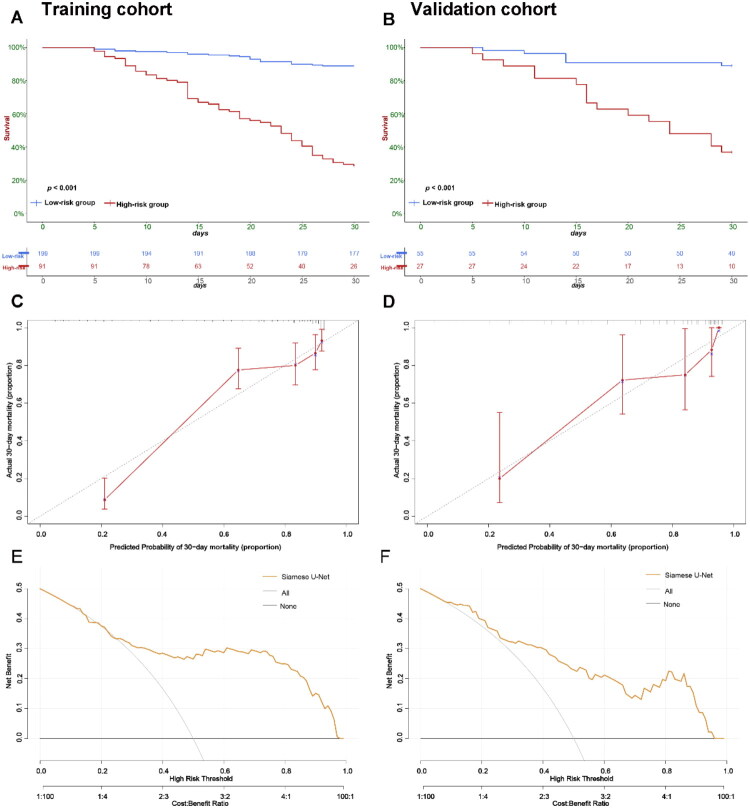
Predictive performance of the Siamese U-Net. Kaplan-Meier curves for 30-day mortality in the training (A) and validation (B) cohorts, stratified by Siamese U-Net-predicted risk (low-risk vs. high-risk groups; *p* < 0.001). Calibration curves of the Siamese U-Net for 30-day mortality prediction in the training (C) and validation (D) cohorts. Decision curves comparing the net benefit of the Siamese U-Net, Treat All, and Treat None in the training (E) and validation (F) cohorts.

For mortality prediction, the Siamese U-Net yielded a C-index of 0.795, an AUC of 0.851, an accuracy of 80%, a sensitivity of 74%, and a specificity of 83%. Using a threshold of 0.4 for the model’s predicted values, significant differences were observed between low- and high-risk subgroups for rates of liver failure, levels of total bilirubin and INR, and 30-day mortality frequency in the validation cohort (*p* < 0.05). Kaplan-Meier survival analysis demonstrated that the model significantly stratified patient groups with distinct 30-day outcomes (*p* < 0.001) (Figure 4A,B). Calibration curves demonstrated close agreement between predictions from the model and observed outcomes (Figure 4C,D). The integrated Brier score for the model was 0.220 in the validation cohort, and DCA showed that the model provided higher clinical net benefit across a broad range of risk thresholds (Figure 4E,F).

### Ablation analysis of the deep learning model

Predictive performance of the ablated models in the training and validation cohorts is detailed in Table S1 and Table 2, respectively. In the validation cohort, the T0-MT-Net achieved a C-index of 0.752 and an AUC of 0.797; corresponding values for the T1-MT-Net were 0.755 and 0.801. The T0-MT-Net and T1-MT-Net showed comparable performance (*p* > 0.05), but both had significantly lower metrics than the Siamese U-Net (*p* < 0.05), suggesting the importance in extracting longitudinal change information. Meanwhile, the Siamese ResNet-101 achieved a C-index of 0.772 and an AUC of 0.822. The Siamese U-Net outperformed the single-task model (*p* < 0.05), highlighting its advantage in the segmentation task. For the Siamese encoder-Net, metrics were 0.770 for C-index, 0.817 for AUC. For the Siamese encoder-Net, performance metrics were 0.770 for C-index and 0.817 for AUC. The Siamese U-Net significantly outperformed the Siamese encoder-Net in predictive accuracy (*p* < 0.01), underscoring its superiority in multi-scale feature extraction.

**Table 2.** Predictive discrimination of the Siamese U-Net and comparisons with ablated models in the validation cohort.

Models	Accuracy	Sensitivity	Specificity	AUC	*p*	C-index	*p*
	(95% CI)	(95% CI)	(95% CI)	(95% CI)		(95% CI)	
T0-MT-Net	76 (65–84)	70 (47–87)	78 (65–88)	0.797 (0.686–0.908)	0.017	0.752 (0.649–0.856)	0.016
T1-MT-Net	77 (66–85)	74 (52–90)	78 (65–88)	0.801 (0.691–0.911)	<0.001	0.755 (0.652–0.858)	<0.001
Siamese ResNet-101	78 (68–86)	70 (47–87)	81 (69–90)	0.822 (0.714–0.929)	0.038	0.772 (0.672–0.872)	0.041
Siamese encoder-Net	78 (68–86)	74 (52–90)	80 (67–89)	0.817 (0.711–0.923)	0.004	0.770 (0.670–0.869)	0.008
Siamese U-Net	80 (70–88)	74 (52–90)	83 (71–92)	0.851 (0.761–0.941)	Reference	0.795 (0.708–0.883)	Reference

### Model visualization and interpretation

**Figure 5.**
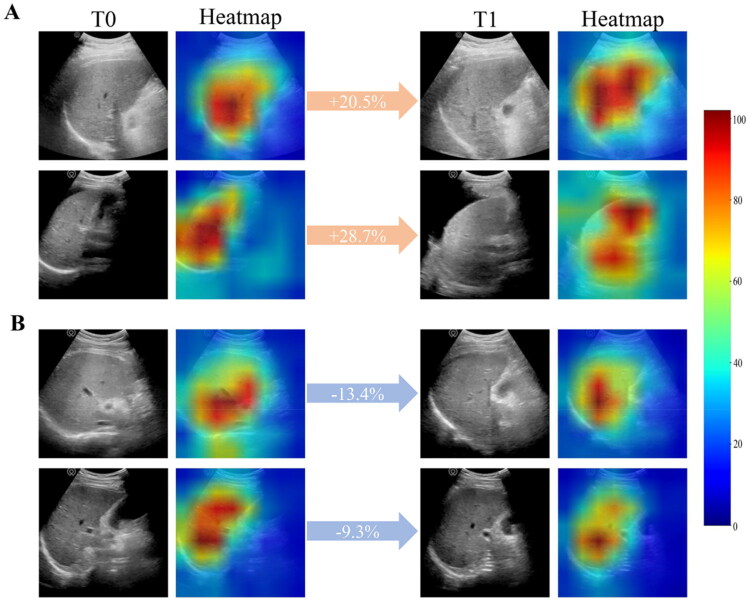
Siamese U-Net visualization and interpretation. Color-coded heatmaps from key channels at the output of the Siamese U-Net, overlaid with corresponding ultrasound images at T0 and T1 time points for four accurately predicted patients. (A) For two non-survivors, T1 heatmap activation intensity percentages were 20.5 and 28.7% higher than T0, with increased activation predominantly in liver texture, portal veins, major vasculature, and ascites. (B) In contrast, T1 heatmap activation intensity percentages for two survivors decreased by 13.4 and 9.3% compared to T0, with reduced activation across hepatic parenchyma and portal venous systems.

Grad-CAM visualizations revealed the model’s layers captured distinct information: shallow layers emphasized basic structural features, including liver morphology, edge characteristics, and vascular patterns, while deep layers extracted high-level semantic features, with activated regions mainly reflecting attention to liver textures, portal venous vasculature, and perihepatic ascites (Fig. S5). Notably, comparison of heatmaps from key channels in the bottom layer of the Siamese U-Net between T0 and T1 images revealed distinct activation trends in survivors versus non-survivor cases. When comparing T0 to T1 images, activation intensities decreased in survivors but increased in non-survivors. Four representative patients (two survivors, two non-survivors) are visualized in Figure 5.

### Integration with clinical parameters

In the training cohort, Cox regression analyses identified total bilirubin, creatinine, and INR as independent clinical predictors (Table S2). Multivariable Cox regression analysis demonstrated that following adjustment for clinical parameters, the Siamese U-Net persisted as an independent prognostic factor for 30-day mortality, exhibiting HRs of 7.9 (95% CI 4.8–12.9, *p* < 0.001) in the training cohort and 4.4 (95% CI 1.6–12.2, *p* = 0.003) in the validation cohort. The combined model achieved a C-index of 0.834, an AUC of 0.892, an accuracy of 85%, a sensitivity of 78%, and a specificity of 85% in the validation cohort. Kaplan-Meier analysis demonstrated that the model distinctly stratified patients by outcomes (*p* < 0.001), while the calibration curve and Brier score of 0.214 indicated good calibration in the validation cohort. DCA showed higher clinical net benefit across risk thresholds (Fig. S6). Predictive performance of the combined model in the training and validation cohorts is detailed in Table S3 and Table 3, respectively.

**Table 3.** Predictive discrimination of the combined model and comparison with clinical model in the training cohort.

Models	Accuracy	Sensitivity	Specificity	AUC	*p*	C-index	*p*
	(95% CI)	(95% CI)	(95% CI)	(95% CI)		(95% CI)	
Clinical model	74 (64–83)	70 (47–87)	76 (63–86)	0.795 (0.693–0.896)	0.003	0.749 (0.653–0.844)	0.006
Combined model	85 (76–92)	78 (56–93)	88 (77–95)	0.892 (0.813–0.972)	Reference	0.834 (0.759–0.910)	Reference

Abbreviations: AUC: area under the curve; CI, confidence interval; C-index, concordance index.

Model comparisons revealed the Siamese U-Net outperformed the clinical model, with significantly higher C-index, AUC, accuracy, sensitivity, and specificity (*p* < 0.01). IDI and NRI showed the Siamese U-Net significantly improved the clinical model (*p* < 0.01), and reduced prediction error rates by 18.3% in the validation cohort. The combined model also outperformed the clinical model significantly (*p* < 0.01), achieving top metrics among all models. IDI and NRI analyses showed that the combined model significantly improved discrimination of the clinical model (*p* < 0.01), reducing error rates by 34.6% in the validation cohort, although it failed to enhance the Siamese U-Net’s performance (*p* > 0.05; Table 4).

**Table 4.** Improvement in predictive performance of the Siamese U-Net and combined model compared with the clinical model.

Model comparison	Training cohort			Validation cohort		
	relative prediction error reduction (%)	IDI	Continuous NRI	relative prediction error reduction (%)	IDI	Continuous NRI
		(95% CI)	(95% CI)		(95% CI)	(95% CI)
Siamese U-Net vs. Clinical model	19.7	0.228 (0.164–0.287)	0.685 (0.569–0.788)	18.3	0.136 (0.041–0.231)	0.413 (0.093–0.694)
*p*		<0.001	<0.001		0.006	0.034
Combined model vs. Clinical model	34.1	0.267 (0.205–0.328)	0.649 (0.548–0.768)	33.9	0.148 (0.054–0.240)	0.403 (0.143–0.705)
*p*		<0.001	<0.001		<0.001	0.004

Abbreviations: CI, confidence interval; IDI, integrated discrimination improvement; NRI, net reclassification improvement.

## Discussion

In this study, we trained a multi-task deep learning architecture capable of leveraging multi-scale imaging features and dynamic patterns extracted from sequential ultrasound images. The proposed Siamese U-Net not only reduces the time required and inter-operator variability for manual segmentation but also enables accurate prediction of 30-day prognosis in HBV-ACLF patients. Moreover, the Siamese U-Net and combined model outperformed the clinical model, demonstrating that deep learning-derived information can effectively complement the limitations of clinical data in prognosis prediction.

In patients with HBV-ACLF, mortality risk changes as hepatic tissue damage exacerbates or resolves during rapid disease progression [11,31]. Prior studies [11,19] reported that time-series clinical data yield more precise predictions than corresponding baseline parameters. Recently, Siamese networks can enhance diagnostic and predictive performance by excelling at capturing subtle differences between input pairs [32,33]. Our comparison of the Siamese U-Net and MT-Nets revealed that the multi-timepoint model outperformed single-timepoint model, underscoring the critical role of longitudinal image analysis in enhancing predictive performance. The HBV-ACLF disease course, characterized by stabilization or deterioration, manifests as pathological changes in longitudinal liver ultrasound images that the Siamese U-Net can quantify but that remain imperceptible to visual inspection. This was also validated by heatmap visualization: activation intensity decreased in survivors and increased in deceased when comparing baseline and early-treatment images.

Although our primary objective was outcome prediction, the Siamese U-Net demonstrated robust liver segmentation capability, consistent with the superior organ-segmentation performance of U-net architectures reported in prior studies [34]. The segmentation precision not only reduces manual delineation time but also enhances predictive performance. Ablation analysis showed that the Siamese U-Net outperformed the Siamese ResNet-101, indicating that the segmentation sub-network is not merely auxiliary but actively strengthens predictive ability. Precise segmentation allows the model to focus on prognostically relevant regions, with boundary information proving valuable for prediction [23,24]. Key-layer heatmaps of the Siamese U-Net corroborated this, highlighting liver morphology, edge characteristics, and perihepatic ascites as critical features.

The Siamese U-Net’s superiority further stems from its integration of multi-scale features (shallow, mid-level, and deep). Model visualization revealed that shallow layers focused on basic structural features, while deep layers extracted high-level semantic features from ultrasound images. Multi-scale feature fusion in deep learning aims to integrate features from different scales, hierarchical levels, and resolutions, enabling models to capture both subtle local details and global structural features [35]. This comprehensive integration facilitates thorough medical image understanding and precise extraction of critical characteristics. In contrast to the Siamese encoder-Net, the prediction sub-network of the Siamese U-Net analyses prognostic information across multi-scale features, achieving superior predictive performance.

Correlation analysis revealed significant associations between the Siamese U-Net’s predictive values and bilirubin/INR levels, which are key parameters for assessing hepatic and coagulation function in liver disease scoring systems [36–38]. These results indicate that the model reflects hepatic pathological changes and functional impairment. Thus, the Siamese U-Ne incorporates comprehensive analysis of multi-level prognostic information, demonstrated superior prognostic performance compared with the clinical model. Additionally, the combined model outperformed the clinical model, achieving the highest efficacy among all evaluated models. The integration of deep learning signature with clinical parameters facilitates comprehensive profiling of HBV-ACLF patients, identifying biomarker abnormalities strongly correlated with prognosis and generating more accurate predictions than clinical-only models. Nonetheless, the combined model failed to significantly enhance the Siamese U-Net may be attributed to the small sample size of the validation cohort and the limited set of clinical indicators available for inclusion.

Several limitations should be acknowledged. First, the sample size was relatively small and retrospectively collected from a single center; future large-scale prospective studies are warranted. Second, we excluded HBV-ACLF patients in intensive care units, which may have introduced selection bias and underestimated mortality rates. Third, unmeasured or inadequately accounted for treatment factors (e.g. antiviral therapy, plasma exchange, albumin dialysis) may have introduced confounding effects, thereby potentially impacting the prognostic accuracy of the Siamese U-Net. Lastly, ultrasound images in this study were sourced from multiple manufacturers, with a subset obtained *via* bedside devices, while factors including liver atrophy, ascites, and bowel gas inevitably compromised image quality. Such quality variability introduced heterogeneity; to mitigate this, future studies will incorporate a larger volume of images from diverse ultrasound devices and protocols to enhance generalizability.

## Conclusion

This study developed a Siamese U-Net for automated liver segmentation and 30-day prognosis prediction in HBV-ACLF patients. The model showed high agreement with manual segmentation, reducing operator variability and time costs. By analyzing multi-scale features and dynamic features in longitudinal ultrasound, it improved the clinical model’s predictive performance. While the Siamese U-Net exhibits promising potential as a non-invasive and precisely accurate tool to facilitate precision management, further large-scale prospective, multicenter validation remains requisite to substantiate its predictive efficacy and generalizability.

## Supplementary Material

Supplemental Material

## Data Availability

Due to the privacy of patients, all datasets generated and analyzed in the current study are not available unless a reasonable request to the correspondence author approved by the institutional review board of the First Affiliated Hospital of Nanchang University (P.X., xupan_1989@126.com).
