# Supplementary material for: Predicting prognosis of patients with hepatitis B virus-related acute-on-chronic liver failure from longitudinal ultrasound images using a multi-task deep learning approach
Source: Ann Med. 2025 Aug 26;57(1):2551819. doi: 10.1080/07853890.2025.2551819 (PMC12381971; doi:10.1080/07853890.2025.2551819)
Supplement: Supplemental Material [file IANN_A_2551819_SM7335.docx]

**Supplementary data**

**S1: Definitions used in the study**

**1. Asian Pacific Association for the Study (APASL) of the Liver acute-on-chronic liver failure (ACLF)**

ACLF was diagnosed according to jaundice (serum bilirubin ≥ 5 mg/dl) and coagulopathy (INR ≥ 1.5 or prothrombin activity < 40%) complicated by ascites or encephalopathy within 4 weeks in a patient with chronic liver disease or cirrhosis.

**2. APASL of the organ involvement[2]**

| Organ/system | Liver (bilirubin, mg/dL) | Renal (creatinine, mg/dL) | Coagulation (INR) | Cerebral (HE grade) | Respiratory (PO2/FiO2) | Circulation (MAP, mm Hg) |
| --- | --- | --- | --- | --- | --- | --- |
| No OF | <5 | <0.7 | <1.8 | Non-HE | >400 | >70 |
| OD | 5-12 | 0.7-1.5 | 1.8-2.5 | I-II | 200-400 | 65-70 |
| OF | ≥12 | >1.5 | >2.5 | III-IV | ≤200 | <65 or vasopressor use |

AARC = APASL ACLF research consortium, FiO2 = fraction of inspired oxygen, INR = international normalized ratio, HE = hepatic encephalopathy, MAP= mean arterial pressure, OD = organ dysfunction, OF = organ failure, PO2 = partial pressure of oxygen.

**S2:** **Liver segmentation**

To train the Siamese U-Net and validate its segmentation performance, two radiologists (X.Z.H. and P.X.) with 8 and 15 years of abdominal ultrasound experience collaboratively selected the matched right intercostal oblique section (first hepatic portal section) from Day 1 (T0) and Day 5 (T1) images for each patient as the target section. Due to its ability to clearly visualize the hepatic anatomical structure, cover a substantial area of liver tissue, offer ease of operation, and its established use in previous studies, the right intercostal oblique section (the section of the first porta hepatis) was selected as the target section. The liver contour was manually delineated as the region of interest (ROI) using ITK-SNAP software version 3.8 (https://www.itksnap.org/). Prior to model training, all images and ROIs were reviewed by a radiologist (A.Y.Z., over 20 years of abdominal ultrasound experience) to ensure matching imaging sections between the T0 and T1 images and accurate segmentation for each patient.

**S3:** **Development of the Deep Learning Model**

**1) Siamese U-net Deep Learning Model (Siamese U-Net) Architecture**

**a. Segmentation Sub-network**

This sub-network features two parallel modified U-nets, each consisting of an encoder (contracting path), decoder (expansive path), and skip connections. At each level of the encoder path, 3×3 convolutions with stride 2 reduce spatial resolution while increasing channel depth. Batch normalization and dropout layers are incorporated to enhance robustness against overfitting. Each level of the decoder path initiates with a transposed convolutional layer to upsample features, which are then concatenated with corresponding encoder-path features via skip connections to preserve spatial context.

**b. Prediction Sub-network**

Designed to leverage multi-scale features, this sub-network fuses features from three hierarchical levels of the segmentation sub-network: (1) the intermediate layer in the contracting path, (2) the bottleneck layer of the U-net, and (3) the element-wise summation module at the expansive path. For dynamic feature analysis, corresponding feature maps at these levels are subtracted to capture inter-image differences. Global average pooling is applied to both concatenated and differenced feature maps to generate compact representations. Outputs from these levels are then concatenated and passed through fully connected layers with dropout, yielding the final mortality prediction probability.

**2) Multi-task Loss Function**

The loss function in this model consists of two components: the Focal Loss and a combined Binary Cross-Entropy (BCE) and Dice Loss.

**a. Focal Loss**

The Focal Loss is designed to address class imbalance by down-weighting inliers (easy examples) and thus focusing more on outliers (hard examples) [25]. The number of 30-day survival patients was twice more than that of mortality in our study; thus, we utilized a modified weighted focal loss to serve as a mortality prediction loss function to mitigate the imbalance during training.

ℒ_mortality-𝑝𝑟𝑒𝑑𝑖𝑐𝑡𝑖𝑜𝑛_=−𝑤_mortality_(1-𝑝)^𝛾^𝑦log(𝑝)-𝑤_survival_𝑝^𝛾^(1-𝑦)log(1-𝑝)

𝑦 denotes the ground-truth (y=0 for survival and y=1 for mortality) and 𝑝 is the predicted probability of the network. The parameter 𝛾 is set to 2. The weight parameters 𝑤_mortality_ and 𝑤_survival_ defined as:

𝑤_mortality_=𝑁_survival_/(𝑁_𝑚_+𝑁_survival_)

𝑤_survival_=𝑁_mortality_/(𝑁_𝑚mortality_+𝑁_survival_)

𝑁_mortality_ and 𝑁_survival_ is the number of mortality and survival patients, respectively.

**b. Combined BCE and Dice Loss**

The second part of the loss function is a combination of BCE and Dice Loss. The Binary Cross-Entropy loss is used for binary classification problems, while the Dice loss is specifically designed for imbalanced pixel-level segmentation[26].

The BCE-Dice Loss can be represented as follows:

ℒ_𝑏𝑐𝑒_𝑑𝑖𝑐𝑒_=𝛼ℒ_𝑏𝑐𝑒_+𝛽ℒ_𝑑𝑖𝑐𝑒_

Where, '𝛼' and '𝛽' are parameters used to balance the contribution of BCE loss and Dice loss. The BCE loss, 'ℒ𝑏𝑐𝑒', is calculated as the binary cross entropy between the true labels and predicted labels. The Dice loss, 'ℒ𝑑𝑖𝑐𝑒', is computed as one minus twice the intersection of true and predicted labels divided by the sum of true and predicted labels. In this study, '𝛼' and '𝛽' are both set to 1, giving equal weight to the BCE loss and Dice loss.

The multi-task loss function in this study is linearly combined through the hyperparameters θ and 𝜇, integrating both the segmentation loss and mortality prediction and in a multi-task loss formulation. This can be mathematically defined as:

ℒ _multi-task_ = θℒ_mortality-𝑝𝑟𝑒𝑑𝑖𝑐𝑡𝑖𝑜𝑛_ + 𝜇ℒ_𝑠𝑒𝑔mentation_

Here, ℒ_multi-task loss_ represents the multi-task loss. In this study, while moratility prediction serves as the main branch, the segmentation loss also plays a crucial role. To balance these components in line with our specific needs, we have set θ=0.2 and 𝜇=1.0. This adjustment makes the network pay more attention to the mortality prediction, while still considering the importance of the segmentation task.

**3) Network Training**

Training was conducted on an NVIDIA GeForce RTX 4090, leveraging the Keras framework with a TensorFlow backend. For training the Siamese U-Net, we employed well-established regularization techniques to mitigate overfitting risk, including data augmentation, batch normalization, dropout, early stopping, and learning rate decay. Hyperparameter tuning was performed by separating the training data into training and validation sets at an 8:2 ratio. An Adam optimizer was used with an initial learning rate of 0.001, applied with a decay factor of 0.5. The batch size was set to 32, dropout rate was 0.5. Data augmentation comprised three transformational strategies: random spatial adjustments (translations, scaling, and rotations within ±10 degrees), random flips (horizontal and vertical axes), and photometric modifications (brightness and contrast adjustments).


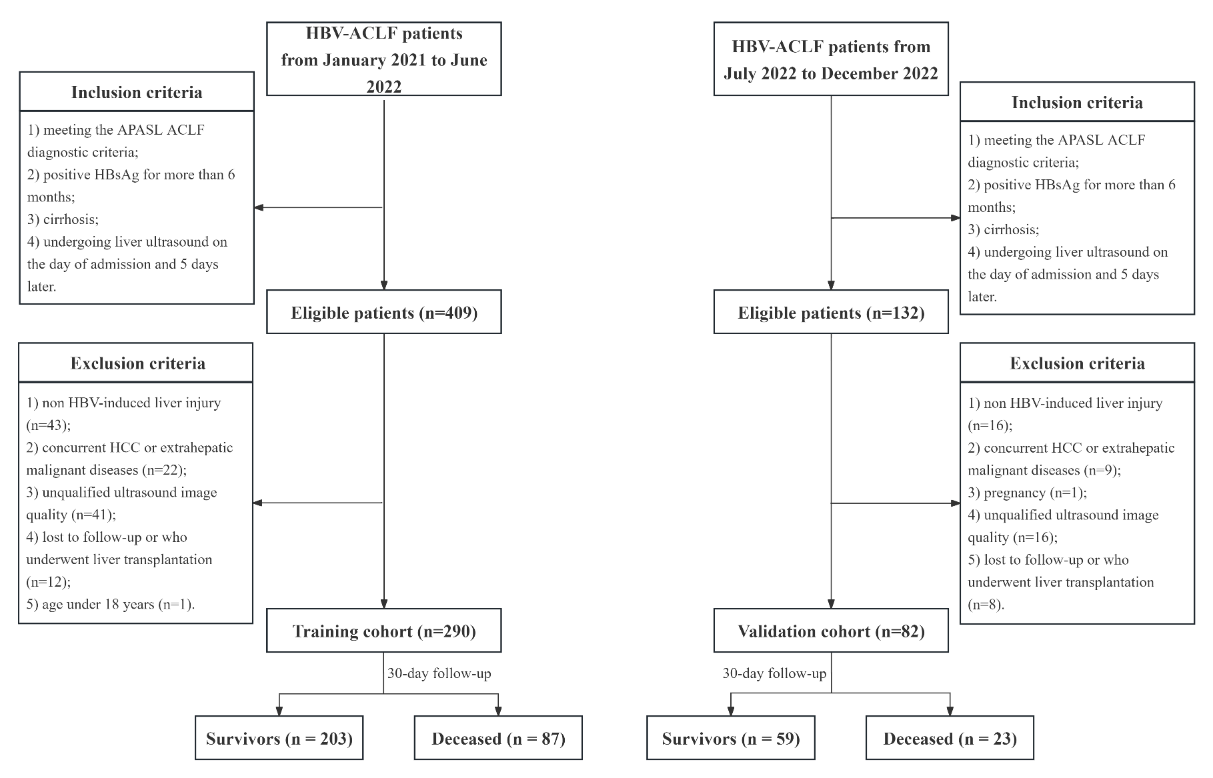


**Supplementary Fig. S1 The flow chart of the study.**

Abbreviations: ACLF, acute-on-chronic liver failure; APASL, Asian Pacific Association for the Study of the Liver; HBV, hepatitis B virus; HCC, hepatocellular carcinoma.


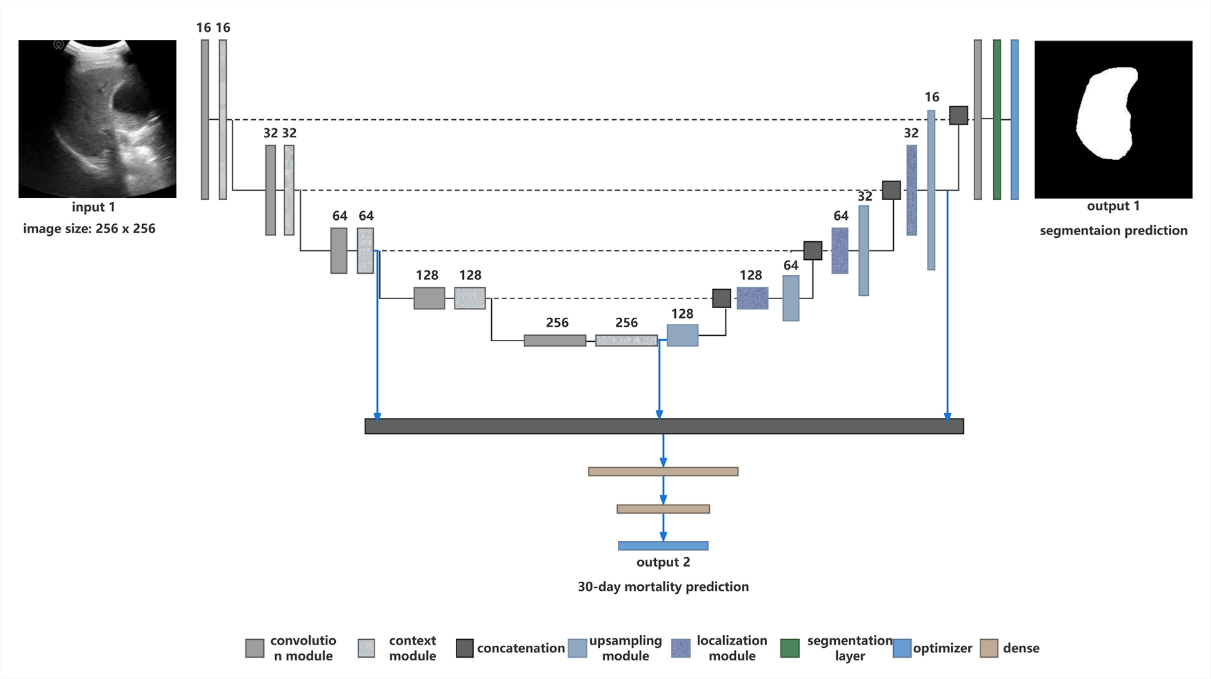


**Supplementary Fig. S2 Detailed illustration of the T0/T1-MT-Net architecture.**

We removed the Siamese network and dynamic information change capture branch but remained a multi-task network for tumor segmentation and mortality prediction in the T0/T1-MT-Net architecture.


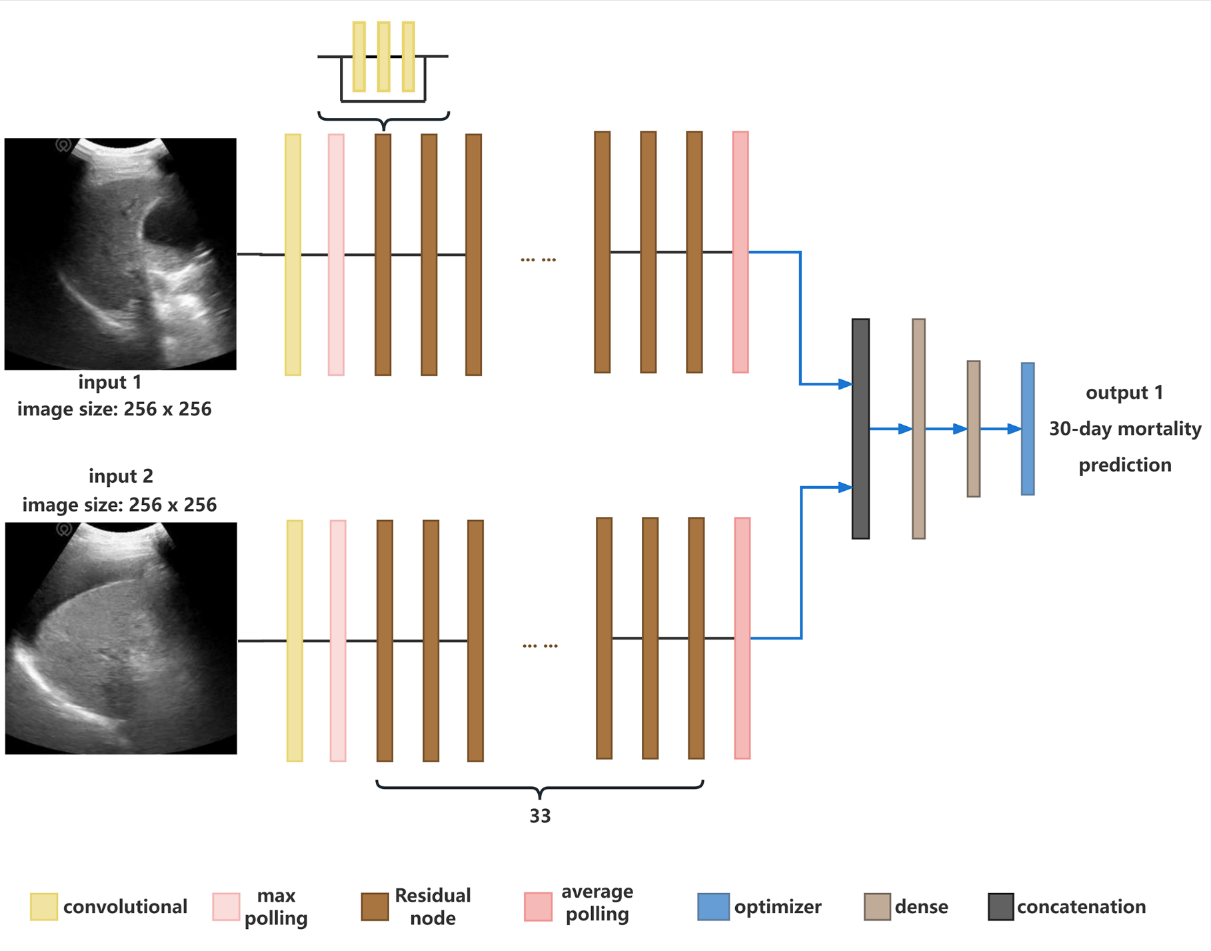


**Supplementary Fig. S3 Detailed illustration of the** **Siamese ResNet-101 architecture.**

We replaced the modified U-Net architecture within the Siamese framework with the ResNet-101 backbone to form Siamese ResNet-101, a single-task mortality prediction.


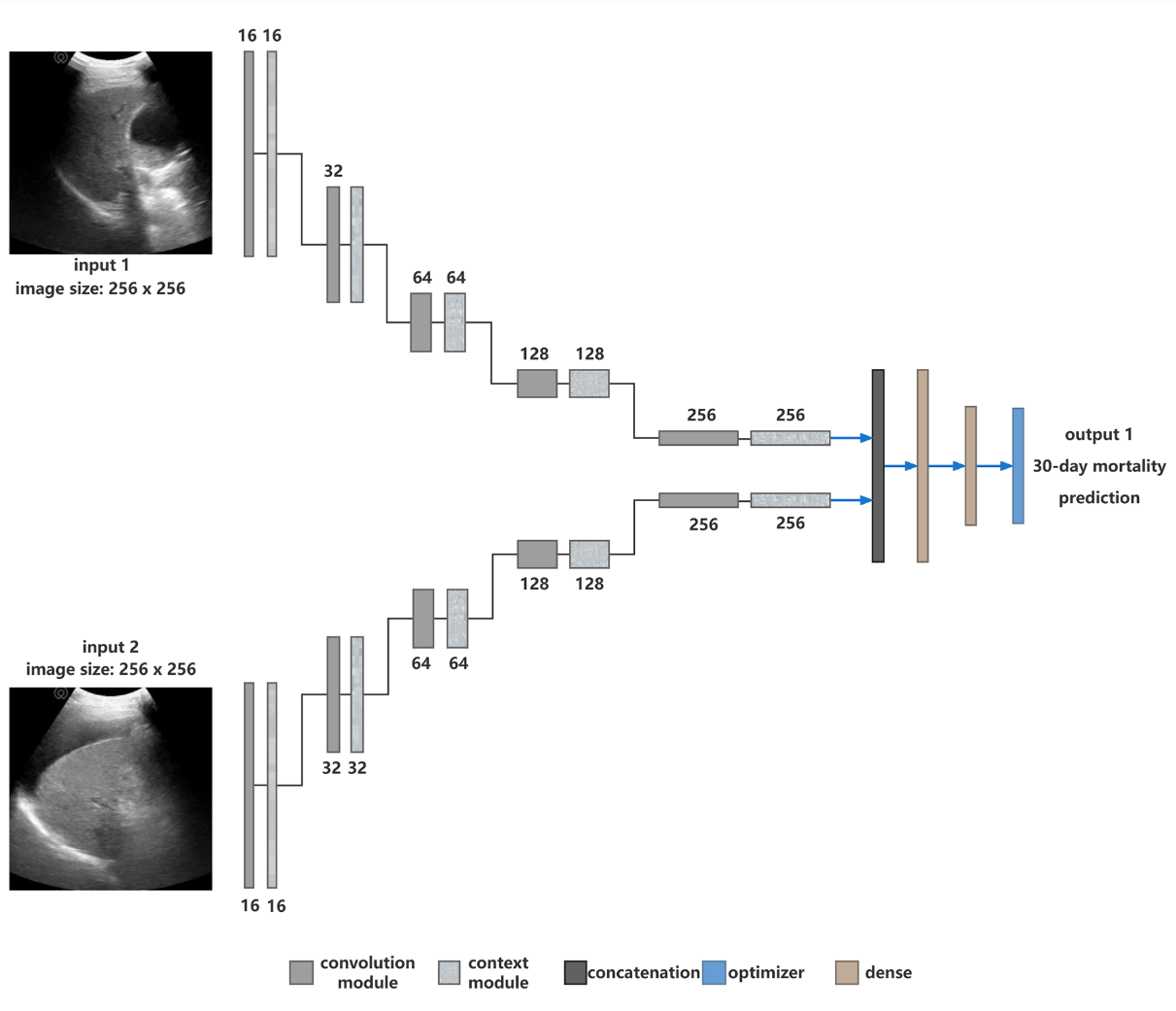


**Supplementary Fig. S4 Detailed illustration of the** **Siamese encoder-Net architecture.**

We removed the decoders and the dynamic information capture branch from the Siamese U-Net to form the Siamese encoder-Net, a single-scale feature-based architecture designed for single-task learning focused on mortality prediction.


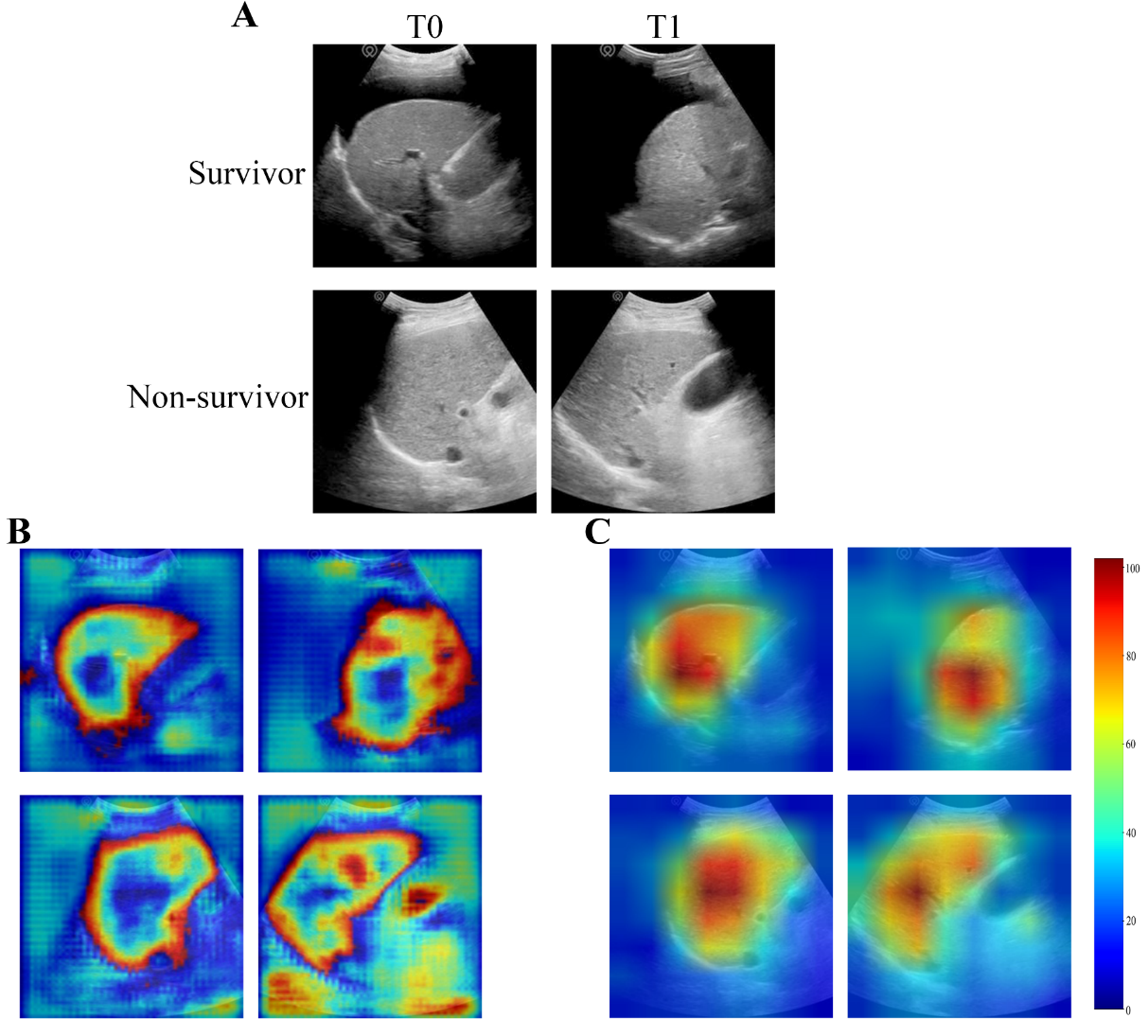


**Supplementary Fig. S5 Longitudinal ultrasound images and corresponding heatmaps generated in Siamese U-Net.**

(A) Longitudinal ultrasound images of patients with different outcomes. (B) Shallow-layer heatmaps, reflect structural features such as liver morphology, edge characteristics, vascular distribution, and overall contour. (C) Deep-layer heatmaps capture high-level semantic features, with activated regions primarily highlighting attention to liver texture patterns, portal venous structures, and perihepatic ascites.

**
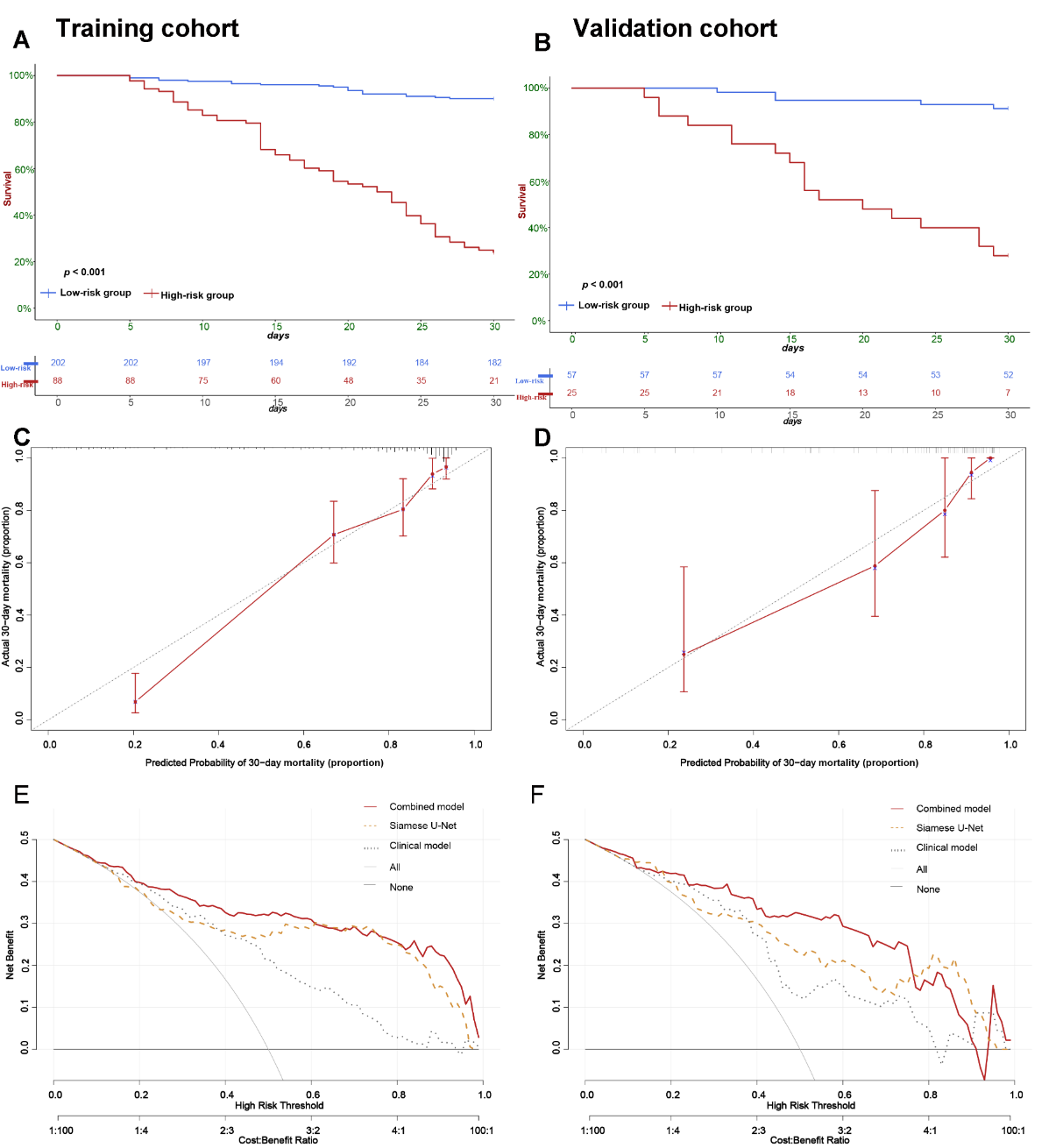
**

**Supplementary Fig. S6 Predictive performance of the combined model.**

(A-B) Kaplan-Meier curves. (C-D) Calibration curves. (E-F) Decision curve analysis.

**Supplementary Table S1 Predictive discrimination of the Siamese U-Net and comparisons with ablated models in the training cohort.**

| Models | Accuracy | Sensitivity | Specificity | AUC | *p* | C-index | *p* |
| --- | --- | --- | --- | --- | --- | --- | --- |
|  | (95% CI) | (95% CI) | (95% CI) | (95% CI) |  | (95% CI) |  |
| T0-MT-Net | 76 (71-81) | 71 (61-80) | 78 (71-83) | 0.798 (0.736-0.860) | <0.001 | 0.756 (0.702-0.810) | <0.001 |
| T1-MT-Net | 77 (72-82) | 72 (61-82) | 79 (73-84) | 0.800 (0.738-0.863) | <0.001 | 0.758 (0.704-0.812) | <0.001 |
| Siamese ResNet-101 | 80 (75-84) | 74 (63-82) | 82 (76-87) | 0.823 (0.772-0.874) | 0.003 | 0.774 (0.729-0.818) | 0.003 |
| Siamese encoder-Net | 79 (74-84) | 74 (63-82) | 81 (75-86) | 0.819 (0.762-0.877) | <0.001 | 0.771 (0.720-0.821) | <0.001 |
| Siamese U-Net | 83 (79-88) | 75 (64-83) | 87 (82-91) | 0.855 (0.802-0.908) | Reference | 0.800 (0.753-0.847) | Reference |

Abbreviations: AUC: area under the curve; CI, confidence interval; C-index, concordance index.

**Supplementary Table S2 Univariate and multivariate Cox regression analyses of independent predictors for 30-day mortality in the training cohort.**

| Patient characteristics | Univariate analysis | | Multivariate analysis | |
| --- | --- | --- | --- | --- |
|  | HR (95% CI) | *p* value | HR (95% CI) | *p* value |
| Age (years) | 1.0 (1.0-1.0) | 0.071 | - | - |
| Female (%) | 0.9 (0.5-1.5) | 0.670 | - | - |
| MAP (mmHg) | 1.0 (1.0-1.0) | 0.850 | - | - |
| HBeAg positive (%) | 0.92 (0.6-1.5) | 0.730 | - | - |
| HBV DNA (log10 IU/mL) | 0.9 (0.9-1.0) | 0.120 | - | - |
| WBC (10^9/L) | 1.1 (1.0-1.1) | 0.078 | - | - |
| Albumin (g/dL) | 0.8 (0.5-1.3) | 0.420 | - | - |
| Total bilirubin (mg/dL) | 1.1 (1.0-1.2) | <0.001 | 1.1 (1.0-1.2) | 0.021 |
| Creatinine (mg/dL) | 1.8 (1.3-2.5) | <0.001 | 1.8 (1.2-2.5) | 0.001 |
| Serum sodium (mmol/L) | 1.0 (1.0-1.1) | 0.710 | - | - |
| ALT (IU/L) | 1.0 (1.0-1.0) | 0.360 | - | - |
| AST (IU/L) | 1.0 (1.0-1.0) | 0.730 | - | - |
| INR | 2.0 (1.6-2.5) | <0.001 | 1.6 (1.2-2.0) | 0.001 |
| AFP (ng/mL) | 1.0 (1.0-1.0) | 0.400 | - | - |
| Siamese U-Net | 10.1 (6.2-16.5) | <0.001 | 7.9 (4.8-12.9) | <0.001 |

Abbreviations: AFP, alpha-fetoprotein; ALT, alanine aminotransferase; AST, aspartate aminotransferase; HBV, hepatitis B virus; CI, confidence interval; INR, international normalized ratio; MAP, mean arterial pressure; PLT, Platelet count; WBC, white blood cell.

**Supplementary Table S3 Predictive discrimination of the combined model and comparison with clinical model in the training cohort.**

| Models | Accuracy | Sensitivity | Specificity | AUC | *p* | C-index | *p* |
| --- | --- | --- | --- | --- | --- | --- | --- |
|  | (95% CI) | (95% CI) | (95% CI) | (95% CI) |  | (95% CI) |  |
| Clinical model | 75 (70-80) | 72(62-81) | 76 (70-82) | 0.795 (0.741-0.850) | <0.001 | 0.751 (0.704-0.798) | <0.001 |
| Combined model | 86(81-90) | 77 (67-85) | 90 (85-93) | 0.898 (0.857-0.940) | Reference | 0.836 (0.798-0.874) | Reference |

Abbreviations: AUC: area under the curve; CI, confidence interval; C-index, concordance index.
